# Study of carbonyl compounds in white wine production

**DOI:** 10.1002/fsn3.1855

**Published:** 2020-09-27

**Authors:** Jakub Herzan, Kamil Prokes, Mojmir Baron, Michal Kumsta, Pavel Pavlousek, Jiri Sochor

**Affiliations:** ^1^ Department of Viticulture and Enology Faculty of Horticulture Mendel University in Brno Lednice Czech Republic

**Keywords:** acetaldehyde, carbonyl compounds, malolactic fermentation, sulfur, sulfur dioxide

## Abstract

Carbonyl compounds, especially acetaldehyde in white wines which have a detrimental effect on the aroma and overall stability of wine, were studied.. Seven wine samples of Grüner Veltliner were produced of one input raw material of grapes, all with different dosage of SO_2_. The sulfur dioxide was maintained at a fixed level during the maturation process and sampled at six months. The grapes were processed, fermented, aged for three months in stainless steel tanks, prepared for bottling, bottled, and then aged in the bottle. In the samples taken, the volume of acetaldehyde, pyruvate, 2‐oxoglutarate, diacetyl, and acetoin was determined by HPLC with diode array detection. Individual forms of SO_2_ were determined by iodometric titration. The wine that was matured on the lees and without the addition of SO_2_ (variant (0/0/0)) contained the lowest amount of all compounds measured. For example, the volume of acetaldehyde for this wine was 2.7 mg/L at the end of the experiment. The results of the sensory analysis showed that such wine could compete with wines with higher SO_2_ content without any problems.

## Graphical abstract

In this study, seven wine samples were produced of one input raw material of grapes, and all seven differed in the dosage of SO_2_. The sulfur dioxide was maintained at a fixed level during the maturation process and sampled at six months. The grapes were processed, fermented, aged for three months in stainless steel tanks, prepared for bottling, bottled, and then aged in the bottle. In the samples taken, the volume of acetaldehyde, pyruvate, 2‐oxoglutarate, diacetyl, and acetoin was determined by HPLC (high performance liquid chromatography) with diode array detection. Individual forms of SO_2_ were determined by iodometric titration.

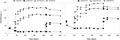

## 1 INTRODUCTION

Sulfur dioxide has been used in winemaking since the late 18th century. Thanks to its properties, such as its action against oxidases, inhibition of microorganisms, and prevention of oxidation, today, we cannot imagine wine made without the addition of SO_2_. Of course, the production of such wine is possible, but its expression is then significantly different from today's modern wines. Apart from its positive effects, SO_2_ can also have its disadvantages. The efficacy of SO_2_ depends mainly on the pH of the wine and the level of phenolic compounds. However, only molecular SO_2_ is active against the growth of microorganisms. In addition, too high a concentration of SO_2_ provides a typical pungent odor and can also cause allergic reactions in some consumers (Vally & Thompson, 2003). That is why the winemaking industry looks for ways to minimize its use (Jackowetz & de Orduña, 2013; Santos, Nunes, Saraiva, & Coimbra, 2013). The difference between free and bound SO_2_ has been known for over a century. First, the difference was attributed to the merging of SO_2_ with carbohydrates, acetaldehyde, and later with other substances. However, there is still little known about the whole process of forming SO_2_ bonds with other substances (Saidane, Barbe, Birot, & Deleuze, 2013).

Among the most important SO_2_ bonds in wines are bonds with carbonyl compounds. These are substances that have one or more aldehyde and ketone functions. It has been shown that ${\text{HSO}}_{3}^{-}$, (molecular SO_2_) is the most reactive form of SO_2_ (Jackowetz & de Orduña, 2013). The largest portion of SO_2_ bound in wine is acetaldehyde. It is an intermediate in the production of ethanol from sugars, produced by decarboxylation of pyruvic acid. It is, therefore, mainly formed in alcohol fermentation. Higher values of acetaldehyde in the wine are observed when sulfuring the must. Acetaldehyde formation is a way to protect the yeast from the antiseptic effects of SO_2_. Another possibility of acetaldehyde formation is chemical oxidation of ethanol during wine storage (Ribéreau‐Gayon, Glories, Maujean, & Dubourdieu, 2006). The highest concentrations of acetaldehyde occur in the presence of free SO_2_ and active yeast. The proportion of acetaldehyde is higher in lengthy fermentations and thiamine deficiency (Bartra, Casado, Carro, Campamà, & Piña, 2010; Jackowetz & de Orduña, 2013).

Pyruvic acid and 2‐oxoglutaric acid also play an important role in the SO_2_ binding. These are secondary products of alcoholic fermentation. The average percentage of pyruvic acid and 2‐oxoglutaric acid in bound SO_2_ is 20.7% and 16.7%, respectively. It is, therefore, interesting to understand the formation and accumulation of these acids during alcohol fermentation. Their largest proportion is formed at the beginning of the fermentation process, and its volume decreases toward the end of the fermentation. Other substances are associated with the accumulation of pyruvic acid. It is a substrate for the formation of acetoin and diacetyl, which also have a carbonyl group and thus also bind SO_2_ (Wells & Osborne, 2012).

An important aspect for making wine at a low or no dose of SO_2_ is the knowledge of the origin and development of the mentioned compounds. Most of these compounds are the product of the metabolism of yeasts or bacteria, and many factors influence their formation and development, such as thiamine content in the must or the presence of SO_2_ and its volume (whether in wine or must) and the associated technology and philosophy of wine production. Understanding the issue of the formation and development of carbonyl compounds can lead to a reduction in total SO_2_ in the final product, that is, bottled wine, without the use of foreign wine substances.

The object of this work is to observe the development of carbonyl compounds in wine production technology with different SO_2_ management.

## 2 MATERIAL AND METHODS

### 2.1 Experimental design

The samples in the experiment come from the same material and went through the same vinification process, with the only difference being the management of the SO_2_ doses. Free SO_2_ and total SO_2_ and individual carbonyl compounds were monitored at all stages of wine production. The production process included processing grapes, maturing wine in stainless steel tanks for three months, preparing wine for bottling (finalization) for about 20 days, and maturing the wine in bottles for one month.

Grapes of the Grüner Veltliner (from the wine region of Moravia, subregion Velkopavlovická, Kobylí village from Czech Republic) were processed in the destemmer. In this operation, the mash was divided into two variants. The first variant was not treated with SO_2_, and the second variant was treated with a dose of 60 mg/L SO_2_. In this step, the first sample of each variant was measured for free and total SO_2_ and carbonyl compounds. This was followed by a one‐day maceration of the mash at 10°C, then pressing and gravity settling using bentonite at a dose of 50 g.hl^‐1^. After the sedimentation of the sludge particles, the must was racked, samples were taken, and the pure yeast culture was fermented.

After the fermentation, the wines were racked from raw yeast sediment and divided into other variants, resulting in seven final variants of the experiment. Their marking is as follows: (0/0/0), (0/0/35), (0/30/35), (0/60/0), (60/0/0), (60/30/35), and (60/60/0). As soon as the variants were split, SO_2_ was added at doses of 0 mg/L, 30 mg/L, and 60 mg/L, which were kept in the wine for three months. For wines that were not dosed with SO_2_, lees stirring was done once a week. Samples for measurement were taken once every 14 days. After each determination of SO_2_, its volume in the wine was adjusted to the specified value. There was no addition of sulfur in (0/0/0), (0/0/35), and (60/0/0) variations. With variations (0/30/35) and (60/30/35), the amount of SO_2_ was adjusted to 30 mg/L. The amount of SO_2_ was adjusted to 60 mg/L with the last two variations (0/60/0) and (60/60/0).

After three months of maturation, the wines were racked from fine yeast sediment, and SO_2_ was adjusted to the bottling values. Variations (0/0/0), (0/60/0), (60/0/0), and (60/60/0) were bottled without SO_2_ addition. For variations (0/0/35), (0/30/35), and (60/30/35), the amount of SO_2_ was adjusted to 35 mg/L. As these values stabilized in the wines, bottling was done. The bottles were labeled and stored in a cellar where the wine matured for the following month at 10–12°C. After this time, sensory analysis of the individual samples was performed. The wine was also evaluated analytically. All samples were frozen to measure the carbonyl compounds that were taken together after the experiment.

### 2.2 Determination of SO_2_ content

The content of free and total SO_2_ was determined by iodometric titration (Joslyn, 1955).

### 2.3 Determination of concentration of carbonyl compounds

The concentration of carbonyl compounds was determined by high performance liquid chromatography (HPLC) with diode array detection to detect and quantify carbonyl compounds in the wine based on the addition of 2,4‐dinitrophenylhydrazines. The method is based on the hydrolysis of carbonyl compounds bound to SO_2_. This technique offers good specificity, repeatability (RSD 0.45%–10.6%), and detection limits (1.29–7.53 µg/L). The total time between the two samples was 22 min. Data in the 200–520 nm range were recorded for 19 min. The chromatogram was scanned at 365 nm.

#### 2.3.1 Instrumentation

Shimadzu LC‐10A Binary High‐Pressure System, Controller system SCL‐10Avp, Two pumps: LC‐10ADvp, Rheodyne Manual Thermostatic Valve Column Thermostat: CTO‐10ACvp, DAD Detector: SPD‐M10Avp, Software: LC‐solution.

#### 2.3.2 Separation conditions

Alltech Alltima C18 3 µm Column; 3 × 150 mm + 3 × 7.5 mm precolumn, Separation temperature 60°C, sample injection volume: 20 µ/L, Mobile phase flow 0.6 mL/min., Mobile phase A: 15 mM HClO_4_, Mobile Phase B: 90% ACN (acetonitrile).

#### 2.3.3 Gradient program

0.00 min: 25% B, 10.00 min: 50% B, 15.00 min: 100% B, 16.00 min: 100% B, 16.01 min: 0% B, 16.49 min: 0% B, 16.50 min: 25% B

### 2.4 Sensory analysis

The wines were evaluated by eight tasters who had certificates of participating in the selection of specialized expert assessors for the sensory analysis of wine, according to ČSN ISO 8586‐1 or ČSN ISO 8586‐2. All variants were assessed using the 100‐point scale according to international union of oenologists IUOE.

## 3 RESULTS AND DISCUSSIONS

The aim of this study was to determine how the amount of SO_2_ added to wine during production affects the formation and development of carbonyl compounds. The results are mapped from grape processing to wine maturation in the bottle over six months (exactly 182 days).

Seven different approaches to wine sulfuring have been chosen for the experiment, which has led to changes in the evolution of carbonyl compounds, thus identifying the critical points of vinification in terms of reducing the use of SO_2_. The determination of carbonyl compounds in wine is complicated because of their instability and the tendency to react reversibly with SO_2_.

### 3.1 Assessment of acetaldehyde

**Figure 1:**
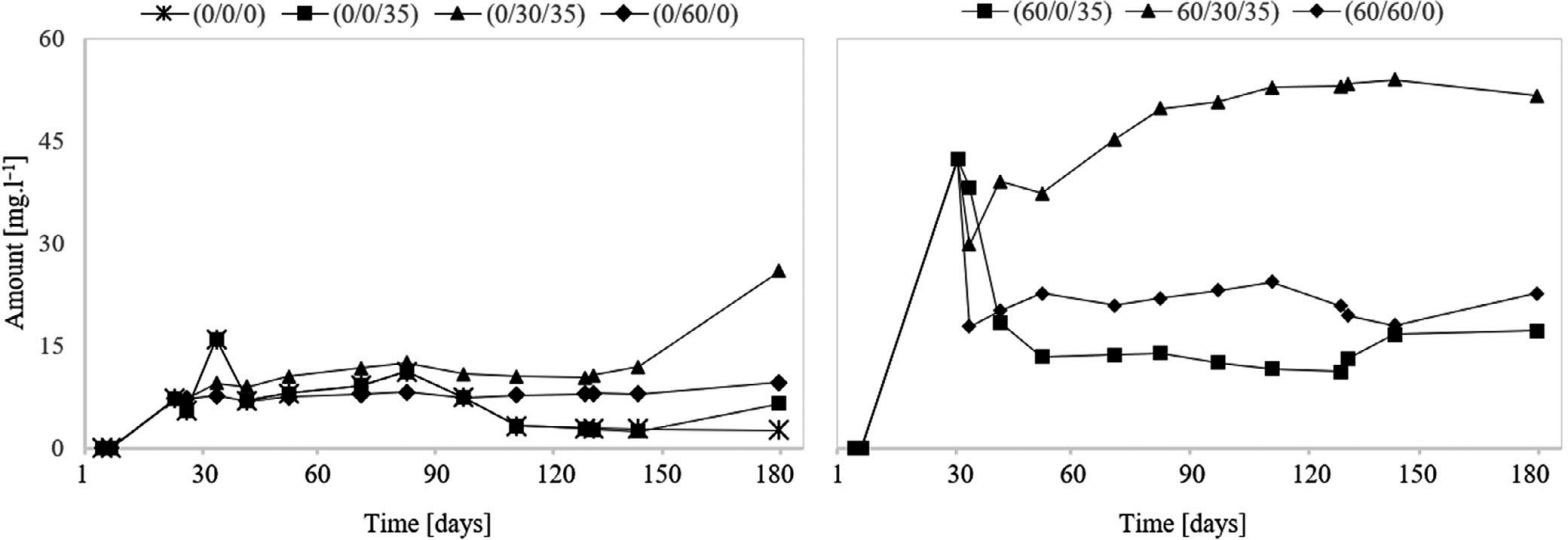
Development of acetaldehyde during the experiment

Ribéreau‐Gayon et al. (2006) reported that the main source of acetaldehyde is alcoholic fermentation. It is an intermediate and is formed by decarboxylation of pyruvic acid. Another source of acetaldehyde can also be a grape attacked by gray mold. When sulfuring such a must, it must be considered that a certain portion of SO_2_ immediately changed to the bound form. The measurement results show that the acetaldehyde content in the wine also significantly affects the must sulfuring before fermentation. In Figure 1, the higher acetaldehyde content of these samples is obvious (variants (60/0/35), (60/30/35), and (60/60/0)). The studies by Bartra et al. (2010) and also Jackowetz & de Orduña (2013) also confirmed this fact. The acetaldehyde content then decreases rapidly within a few days after fermentation, except variant (60/30/35), where the amount of acetaldehyde increased. In this variant, SO_2_ was maintained at 30 mg/L after fermentation. An increased amount of acetaldehyde, compared to other nonsulfured samples before fermentation ((0/0/0), (0/0/35), and (0/60/0)), can also be observed in variant (0/30/35), which, after fermentation, also had a level of free SO_2_ maintained at 30 mg/L. Thus, in order to minimize acetaldehyde, a dose of 30 mg/L is not sufficient after the end of fermentation. For the same reason, it is also not advisable to sulfurize the must before fermentation. If our aim is to reduce the acetaldehyde content of the wine to its lowest level, it is recommended to exclude the use of SO_2_ not only before fermentation but also during the first months of vinification. Preventing the oxidation of wine will ensure occasional lees stirring. The measurement results for variant (0/0/0), in which the wine matured in contact with the yeast sediment and without the use of SO_2_, confirm this fact. At the end of the experiment, the acetaldehyde content of this variant was the lowest at 2.7 mg/L. Also, variant (60/0/35), which was not sulfurized during the three‐month maturation, showed the lowest values of acetaldehyde (17.2 mg/L) from the group of nonsulfurized variants before fermentation. Wines aged at 60 mg/L of free SO_2_ (variants (0/60/0) and (60/60/0)) also show a relatively low level of acetaldehyde at the end of the experiment. However, this approach cannot be recommended to reduce the need for wine propagation. These are variants with the highest values of total SO_2_.

The coupled co‐oxidation of ethanol in the presence of atmospheric oxygen leads to chemical formation of acetaldehyde in wines (Danilewicz, 2003; Elias & Waterhouse, 2010). Late alcoholic phase (Jackowetz, Dierschke, & de Orduña, 2011) observed that yeast were able to reutilize acetaldehyde rapidly in the second fermen yeast metabolism contributes to a significant decrease in acetaldehyde levels. Following alcoholic fermentation, contact with yeast lees further reduced acetaldehyde levels. Longer yeast lees contact leads to a continual decrease in acetaldehyde levels from 27 mg/L to 21 mg/L in cider over a 15‐month period (Madrera, Hevia, García, & Valles, 2008). The reduction of acetaldehyde during MLF is also significant (Osborne, Mira de Orduna, Pilone, & Liu, 2000). Postfermentative vinification stages contributed significantly to de novo acetaldehyde formation. Aging and bottling operations represent critical control points, with some contribution from filtration. This knowledge may allow to reduce both acetaldehyde and SO_2_ levels.

### 3.2 Assessment of pyruvate

**Figure 2:**
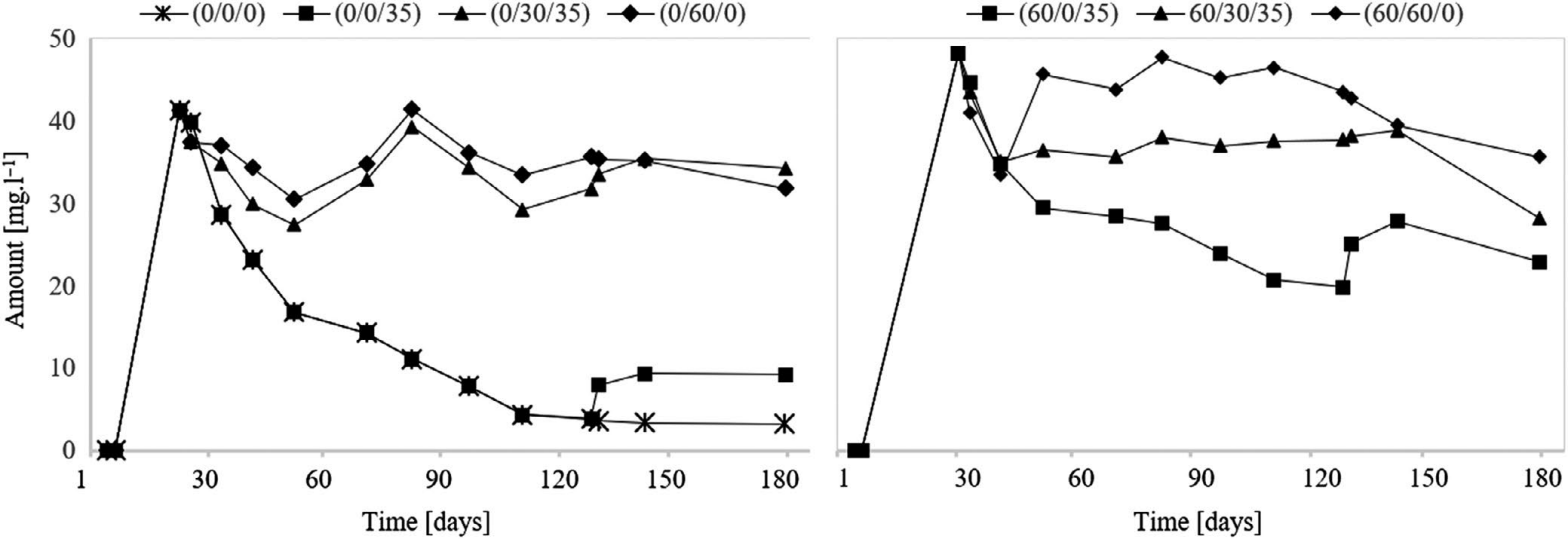
Development of pyruvate during the experiment

The development of pyruvate in individual variants is shown in Figure 2. In the case of wine without SO_2_ (variant (0/0/0)), it is possible to observe a gradual decrease in the pyruvate content in the wine. During the experiment, pyruvate decreased in this variant to 3.2 mg/L. Wells & Osborne (2012) reported that pyruvate is a substrate for the formation of acetoin and diacetyl during malolactic fermentation which in variant (0/0/0) has occurred and is, therefore, the cause of the reduction in pyruvate to its lowest value. The presence of free SO_2_ prevents the development of lactic acid bacteria, thereby contributing to a higher amount of pyruvate in the wine. A gradual decrease in pyruvate can also be seen in variant (60/0/35) that was not sulfurized during the three‐month maturation. Sulfur dioxide was used during the finalization of the wine when the pyruvate level increased again. Thus, it can be stated that the use of SO_2_ immediately after fermentation will cause a high amount of total SO_2_ content in the wine, the high percentage of which will represent SO_2_ bound to pyruvate. If the first application of SO_2_ occurs at least 60 days after the fermentation, the SO_2_ will not go into the bound form so much, and thus, its dosage can be reduced (Figure 2).

The study by Jackowetz et al. (2011) determined pyruvic acid levels in range 5–92 mg/L in white wines, which are higher than in this work. Earlier study by Rankine (1968) observed values from 21 to 147 mg/L in laboratory fermented commercial juices.

Schwinn, Durner, Delgado, & Fischer (2019) studied effect of stirring in tanks on the production of pyruvic acid and acetaldehyde by yeast after inoculation. They found the higher concentration of pyruvate between the third and sixth day after the inoculation in unstirred tank, in total concentration up to 190 mg/L. In stirred tank were found production of pyruvate on the sixth day, but in lower total concentration (up to 60 mg/L). These big differences were caused by different nutrition levels at the top and the bottom of tanks. Production of acetaldehyde was not affected, and the higher concentration was observed at the sixth day after inoculation (≤60 mg/L).

Yeast pyruvate formation may be influenced by the nutritional status of the musts. In sweet French wines supplemented with thiamine, pyruvate concentrations were determined in range ≤51 mg/L, but in the same wines without nutritional supplementation, pyruvic acid levels were found in concentration up to 330 mg/L (Ribéreau‐Gayon, Dubourdieu, Donèche, & Lonvaud, 1998; Ribéreau‐Gayon, Glories, Maujean, & Dubourdieu, 1998). Thiamine pyrophosphate is an essential cofactor for pyruvate decarboxylase (Pronk, Yde Steensma, & van Dijken, 1996; Ribéreau‐Gayon et al., 2006). Whiting (1976) reported that a lack of thiamine pyrophosphate, and hence excessive pyruvate inside the yeast cell, can be the reason for pyruvate excretion at a concentration greater than 100 mg/L.

White wines usually do not undergo malolactic fermentation (MLF) that typically occurs after alcoholic fermentation, resulting in the decarboxylation of L‐malic to L‐lactic acid and wine deacidification. Malolactic fermentation is carried out by wine lactic acid bacteria, which are known to degrade some carbonyls including pyruvic acid and acetaldehyde (Flamini, De Luca, & Di Stefano, 2002) in addition to malic acid.

### 3.3 Assessment of 2‐Oxoglutarate

**Figure 3:**
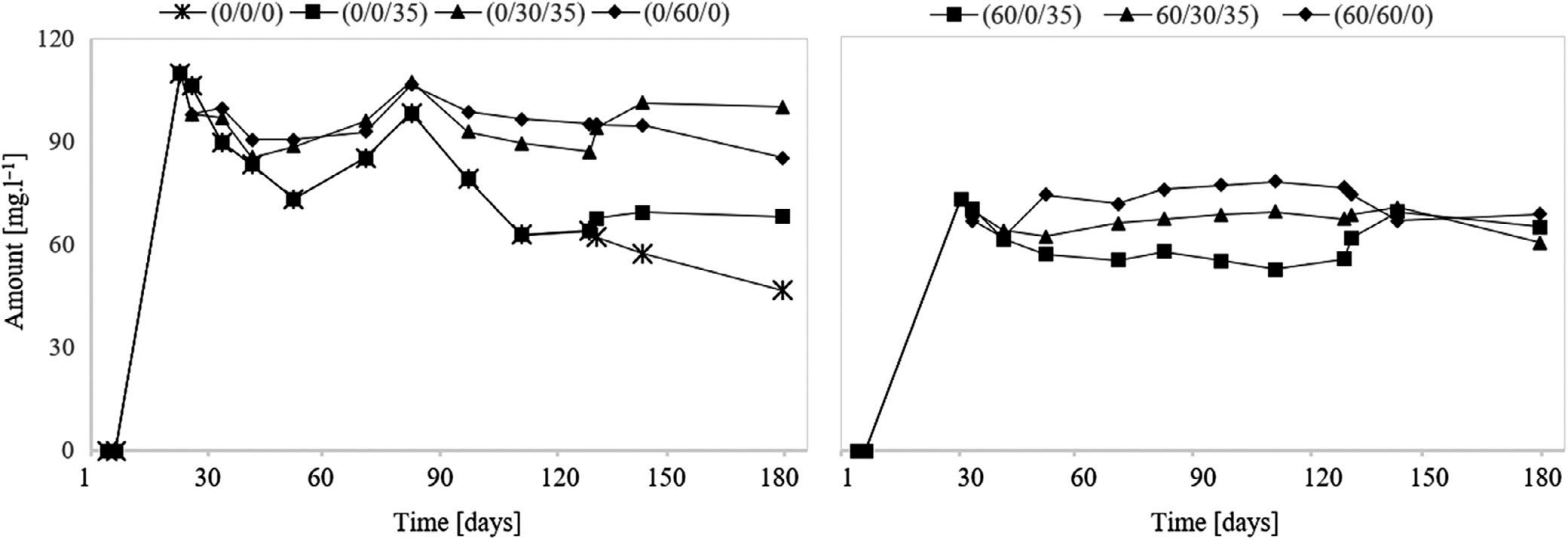
Development of 2‐oxoglutarate during the experiment

The volume of 2‐oxoglutaric acid is almost 40 mg/L in samples from unsulfurized must compared to the samples that were sulfurized before fermentation. Thus, the presence of SO_2_ during fermentation causes the yeast to produce less 2‐oxoglutarate. During the aging of the wine, the value of 2‐oxoglutarate is relatively stable when the must is treated with SO_2_. Conversely, in the absence of SO_2_ during fermentation, the concentration of 2‐oxoglutarate is higher, and its content varies considerably during the aging of the wine. The development of 2‐oxoglutarate is also affected by the level of SO_2_ with which the wine matures. The results show a certain correlation between the amount of SO_2_ present during the aging of the wine and the amount of 2‐oxoglutarate. If the wine matures without the addition of SO_2_ (variants (0/0/0), (0/0/35), and (60/0/35)), the level of 2‐oxoglutarate decreases. As soon as the wine is sulfurized, the amount of 2‐oxoglutarate in the wine will increase (Figure 3).

The study by Jackowetz & de Orduña (2013) determined concentration of 2‐oxoglutarate in range 6–202 mg/L (31 mg/L average) in different table white wines.

Earlier different studies found similar concentration range, for example in sweet dessert wines, concentration ranges of 70–273 mg/L (Ribéreau‐Gayon et al., 2006; Ribéreau‐Gayon, Dubourdieu, et al., 1998; Ribéreau‐Gayon, Glories, et al., 1998) and 78–248 mg/L (Barbe, de Revel, Joyeux, Lonvaud‐Funel, & Bertrand, 2000) have been reported. Rankine (Rankine, 1968) reported an average of 53 mg/L with a range of 6–135 mg/L in Australian white wines.

### 3.4 Assessment of diacetyl and acetoin

**Figure 4:**
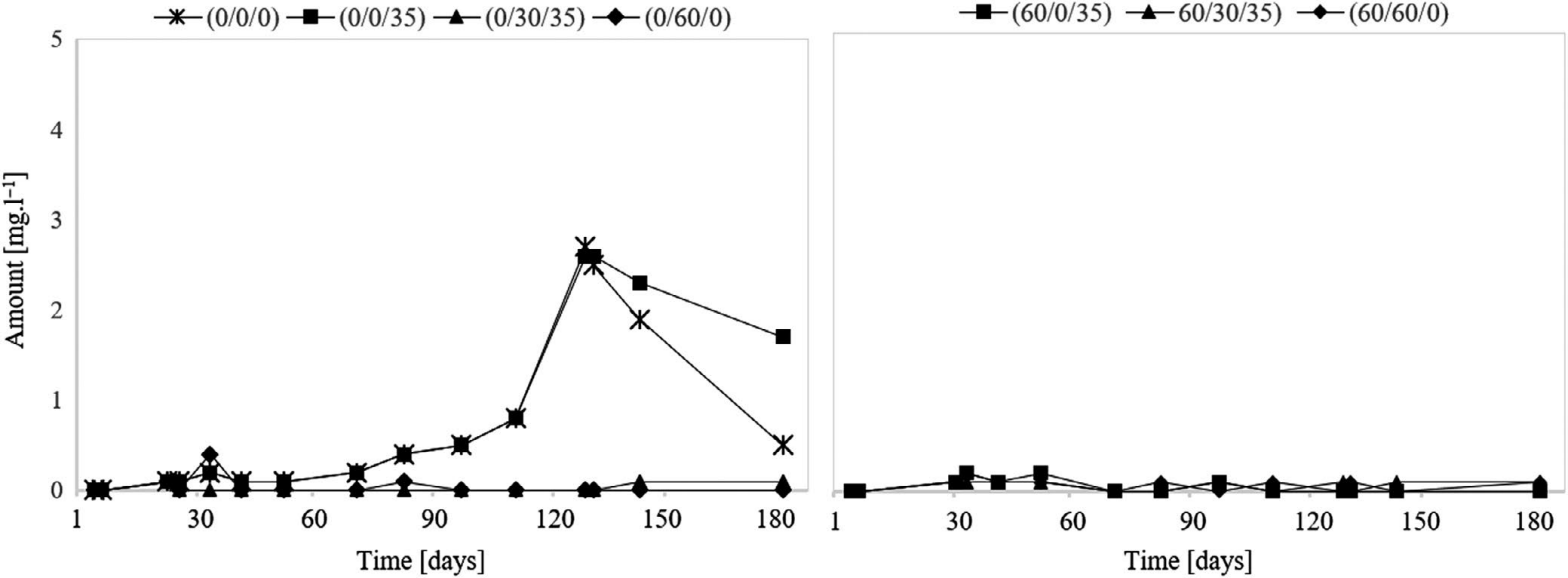
Development of diacetyl during the experiment

**Figure 5:**
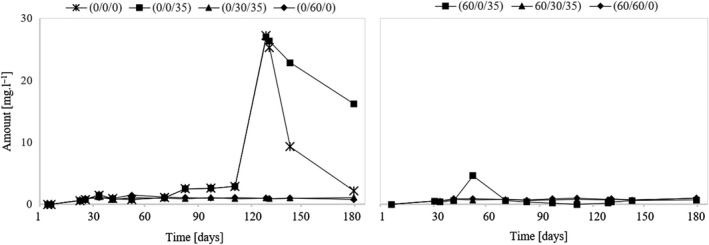
Development of acetoin during the experiment

Viljakainen and Laakso (Viljakainen & Laakso, 2002) reported that lactic acid bacteria process citric acid in part to acetic acid, but also diacetyl and acetoin. The presence of diacetyl and acetoin was detected only in variants that were not sulfurized before fermentation and subsequently during the three‐month aging of the wine (see Figure 4 and 5, variants (0/0/0) and (0/0/35)), which demonstrates the activity of lactic bacteria and their inhibition by free SO_2_. The MLF did not run in variant (60/0/35), although it also matured for three months without using SO_2_. This demonstrates that sulfur dioxide present in the wine only in bound form is sufficient to inhibit lactic acid bacteria. From this fact, it can be concluded that by sulfurizing the must it is possible to preclude the premature course of the malolactic fermentation. Furthermore, the results show that the acetoin and diacetyl content decrease during the aging of the wine. If SO_2_ is applied immediately after the MLF is terminated, that is, when the diacetyl and acetoin contents are highest, their volume decreases significantly and more slowly than if the sulfuring had never happened. As shown by the results of variant (0/0/0), the amount of diacetyl and acetoin is reduced to nearly zero over approximately two months, which is crucial in minimizing an unwanted butter tone in the wine caused by diacetyl and acetoin.

### 3.5 Assessment of free SO_2_

**Figure 6:**
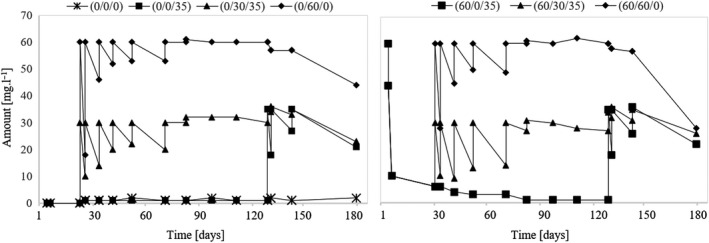
Development of free SO_2_ during the experiment

The development of free SO_2_ during the experiment is shown in Figure 6. Samples that were sulfurized before fermentation (variants (60/0/35), (60/30/35), and (60/60/0)) reported a decrease in free SO_2_ to almost zero during fermentation. This is evidence of the production of acetaldehyde by yeast as a defense against the antiseptic action of free SO_2_. The presence of free SO_2_ also caused a lengthy and problematic fermentation process in the mentioned variants, which harmed the resulting wine aroma.

In the case of using SO_2_ immediately after fermentation (variants (0/30/35), (0/60/0), (60/30/35), and (60/60/0)), the level of free SO_2_ decreases due to the presence of carbonyl compounds that react with the free SO_2_ to form carbonylsulfuric acids (Ribéreau‐Gayon et al., 2006). The SO_2_ level must be increased again to the required value to prevent possible oxidation or degradation of the aroma of the wine. This step should be repeated until the SO_2_ level has stabilized to the desired value. As shown in Figure 6, the SO_2_ level stabilized after six weeks of redosing of all experimental varieties. It is also apparent from the figures that the decrease in free SO_2_ is more pronounced in variants that were already sulfurized before fermentation (variants (60/0/35), (60/30/35), and (60/60/0)), which again points to a higher concentration of carbonyl compounds and thus lower wine stability in view of maintaining the desired level of free SO_2_.

### 3.6 Assessment of total SO_2_

**Figure 7:**
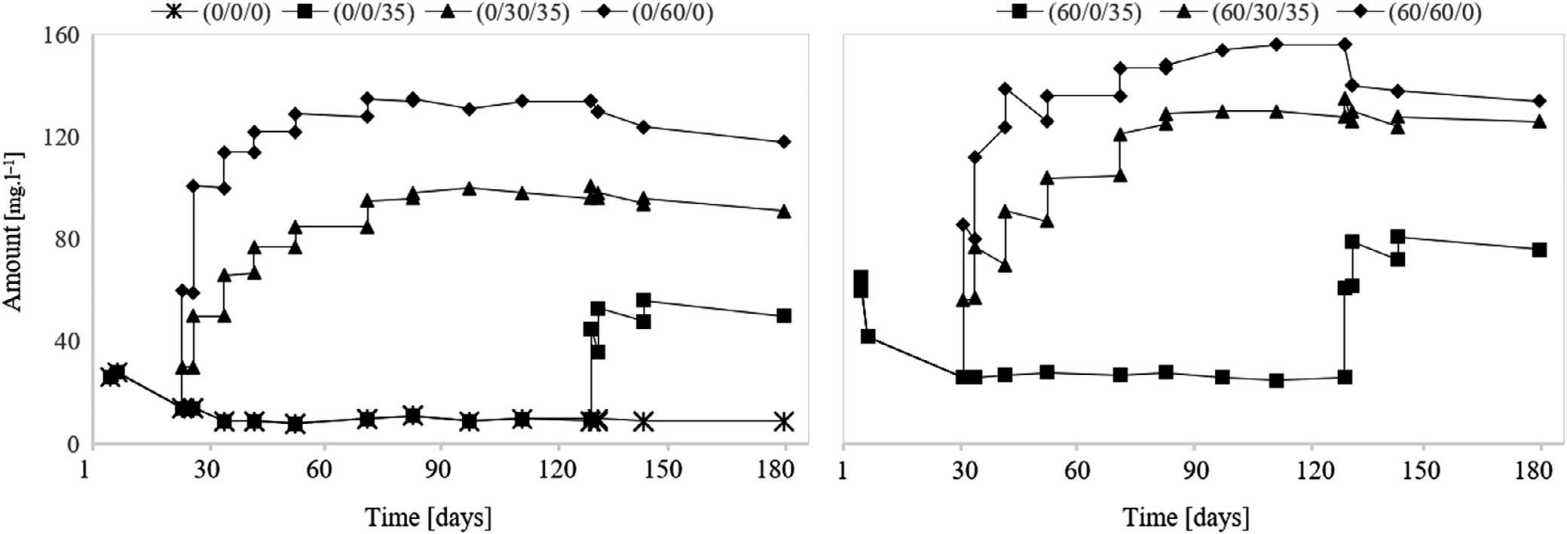
Development of total SO_2_ during the experiment

Total SO_2_ is the so‐called memory of wine development. It indicates the quality of grapes and subsequent oenological work Saidane et al. (2013). As can be seen in the graph of the development of all SO_2_ (Figure 7), its content is lower than the legal maximum allowed for all variants. Nevertheless, there are significant differences in the amount of total SO_2_ between the variants, even though the wines are made from the same material.

For all variations of the experiment, it is possible to observe a decreasing level of total SO_2_ during the vinification process that does not match the amount of SO_2_ added to the wine. For example, variant (60/60/0), which was treated with the highest total amount of SO_2_, 189 mg/L, during the vinification process contains only 134 mg/L at the end of the experiment. The same trend can be observed for all variants. For variants sulfured before fermentation (see Figure 7 variants (60/0/35), (60/30/35), and (60/60/0)), this loss is on average 30%, and for variants unsulfured before fermentation (see Figure 7 variants (0/0/0), (0/0/35), (0/30/35), and (0/60/0)), the loss is only 15% on average. Thus, it can be stated that the amount of SO_2_ used does not correspond to the amount of total SO_2_ that will be measured in the wine. The graphs also show the following. Although yeast produces so‐called endogenous SO_2_, the level of all SO_2_ measured is lower in all variants after fermentation than before the fermentation.

The most important carbonyl SO_2_ binders in white table wines were calculated as being acetaldehyde, followed by pyruvic and 2‐oxoglutaric acid. **T**hese compounds have some of the lowest dissociation constants of quantitatively important wine carbonyls. In red table wines, 2‐oxoglutaric acid was found to be more relevant for SO_2_ binding, and the weight of galacturonic acid was similar to pyruvic acid. Accordingly, studies aimed at reducing SO_2_ binding in reds should focus on the role of skin maceration and its effects on these compounds (Jackowetz & de Orduña, 2013).

Lajin & Goessler (2019) determined major sulfur compounds by HPLC in white and red wines. The major sulfur compounds were found to be sulfate (50–81 mg/L) followed by sulfite (18–24 mg/L free sulfite and 41–63 mg/L of total sulfite after base hydrolysis). They also detected small amounts of methionine in wine (0.5–1.0 mg/L); they found also a few unknown compounds (collectively 1.0–2.0 mg/L) were observed in the chromatograms, and the sum of detected species accounted for only 65%–77% of total sulfur concentration (105‐165mg/L).

### 3.7 Comparison of the development of available binding SO2 in individual variants

**Table 1:** Comparison of the development of SO_2_‐binding compounds in individual variants

	2‐oxoglutarate (mg/L)	Pyruvate (mg/L)	Acetoin (mg/L)	Acetaldehyde (mg/L)	Diacetyl (mg/L)
(0/0/0)	46.7	3.2	2.2	2.7	0.5
(0/0/35)	68.2	9.2	16.2	6.5	1.7
(0/30/35)	100.2	34.2	1.1	25.9	0.1
(0/60/0)	85.3	31.8	0.8	9.6	0
(60/0/35)	64.7	22.9	0.7	17.2	0
(60/30/35)	60.2	28.2	1	51.6	0.1
(60/60/0)	68.4	35.6	1	22.6	0.1

The table shows the measured values of carbonyl compounds for individual variants in the sample measured at the last sampling, that is, on the day when the sensory analysis of wines was performed.

### 3.8 Sensory analysis

The experiment was concluded with the sensory evaluation of individual variants. The tasting was attended by ten wine tasters. The wines produced from unsulfured must scored higher than those from sulfured must. The beginning of fermentation was suppressed due to the presence of free SO_2_ in the must. This problem manifested not only in the higher content of acetaldehyde in young wine but also by the formation of sulfate notes during the fermentation, which was negatively reflected in the resulting wine aroma.

**Figure 8:**
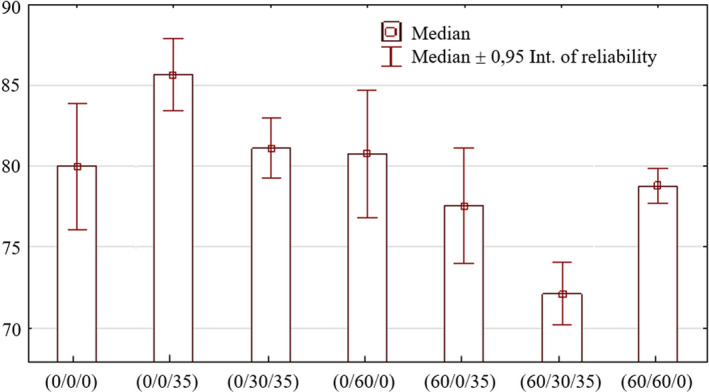
Statistical evaluation of the results of wines evaluated by the 100‐point system

Figure 8 shows the statistically significant difference between variants (0/0/35) and (60/30/35), (0/0/35) and (60/60/0), (0/0/35) and (60/0/35), and (0/0/35) and (0/30/35). Conversely, a statistically significant difference was not found in variants (0/30/35), (0/30/60), (60/60/0), or (0/0/0). These facts show that wines that were unsulfured during winemaking are comparable to those in which SO_2_ was used during production.

Sulfur dioxide (SO_2_) is the most commonly used additive in wineries to limit the production or accumulation of aldehyde compounds in wine (Decker, Elias, & McClements, 2010; Laurie & Clark, 2010; Laurie et al., 2010). Aldehyde compounds are prone to nucleophilic attack by hydrogen sulfite and hence readily form addition products that are odorless (non‐volatile) (Ugliano, 2013). However, as SO_2_ can be gradually depleted during aging (Ebeler, Sacks, Vidal, & Winterhalter, 2015), the addition products can progressively dissociate and release free aldehyde compounds and the accompanying off‐flavors. In contrast to the aldehyde compounds, low molecular weight sulfur compounds can contribute significant “reductive” off‐flavors to wine (Smith, Bekker, Smith, & Wilkes, 2015). Sulfur‐containing pesticides and gaseous sulfur dioxide (SO_2_) or potassium metabisulfite (PMS) added after harvest are potential precursors for low molecular weight sulfur compounds. Grape juice with high turbidity and/or a lack of sufficient oxygen and nitrogen supply during fermentation also facilitates the production of these compounds. For example, hydrogen sulfide (H_2_S), a detrimental low molecular weight sulfur compounds in wine, can be generated by *S. cerevisiae* from elemental sulfur, sulfate, or sulfite through the sulfate assimilation and reduction pathway.

## 4 CONCLUSION

Based on the results obtained, it can be generally said that the application of SO_2_ soon after the end of yeast or bacteria activity leads to an increase in the amount of bound SO_2_. The highest content of carbonyl compounds is present in the wine just after the biological processes have ceased, as they are secondary products.

The results of the experiment show that the development of carbonyl compounds is influenced mainly by the SO_2_ dosing or by the chosen wine production technology. Carbonyl compounds are predominantly a product of yeast and bacterial metabolism. Therefore, their highest content in wine occurs immediately after fermentation. When measuring samples taken before fermentation, zero acetaldehyde was determined in each of the variants as well as other carbonyl compounds, confirming the perfect health of the grapes. The experiment confirmed the fact that the addition of SO_2_ to the grapes (must) increased production of acetaldehyde by the yeast. Acetaldehyde formation is a way of protecting yeast from the antiseptic effects of SO_2_. Furthermore, it has been shown that during the aging of the wine, the content of carbonyl compounds decreases, and, conversely, the content of these compounds increases during the operations like racking or bottling. For wines that were not sulfurized during vinification, where only the fine yeast sediment was stirred, the carbonyl content was reduced to the lowest values. It is clear, therefore, that when the wine is made with sur‐lie technology, SO_2_ is stable during the sulfuring of such wine and does not pass so much into bound form.

Sensory analysis of wines has shown that the amount of free SO_2_ in which the wine matures also has an impact on its aromatic and flavor profile. Therefore, each winemaker should know much earlier than during the processing of grapes, what type of a wine he wants to produce. This decision must be adapted not only to the management of the vineyard work and the timing of the harvest but also to the method of working with SO_2_. This substance is often used by winemakers as if it was crucial to use it. With its responsive and targeted use, one material can produce wine that is, on the one hand, very structural, complex, with a massive body and aroma. On the other hand, the same material can produce wine that is lighter with an expressive secondary aroma that is easy to drink and pleasantly fresh. Winemaking technology and methods, such as sur lie and MLF, that complement and build on each other are probably the most effective tools for reducing SO_2_ in wine. Of course, this is only where it is appropriate for the type and style of the resulting wine.

## 5 INFORMED CONSENT

Written informed consent was obtained from all study participants.

## CONFLICT OF INTEREST

The authors declare that they do not have a conflict of interest.

## ETHICAL STATEMENTS

This study does not involve any human or animal testing.
